# Supplementary material for: DNA Synthesis Is Required for Reprogramming Mediated by Stem Cell Fusion
Source: Cell. 2013 Feb 14;152(4):873–83. doi: 10.1016/j.cell.2013.01.012 (PMC3605571; doi:10.1016/j.cell.2013.01.012)
Supplement: Document S1. Tables S1 and S2 [file mmc1.pdf]

Table S1. Pluripotent Hybrid Colony Formation of Cell-Cycle-Enriched mESCs Fused with mB Cells, Related to Figure 2

	Number of AP^+ $Puro^+$ colonies per 10^7 fused cells				
	G1 (F8)	S (F12)	S/G2 (F13)	G2/M (F16)	Total
Exp. 1	178	1000	n/s	1609	880
Exp. 2	102	n/s	743	526	257
Exp. 3	75	106	276	280	140

Hybrid reprogramming was assessed on the basis of both alkaline phosphatase expression and Puromycin resistance (AP^+, $Puro^+$), 12 days after fusion as described in Figure 2A. n/s indicates not scored.

Table S2A. Percentage of Cells with Different Oct4 Signal Intensity Levels, Related to Figure 3

	Low	Medium	High
G1 (F8)	60	26	14
G1 (F9)	66	24	10
S (F12)	60	30	10
S/G2 (F13)	47	39	15
G2/M (F16)	34	48	18

Table S2B. Percentage of Cells with Different Sox2 Signal Intensity Levels, Related to Figure 3

	Low	Medium	High
G1 (F8)	40	46	14
G1 (F9)	35	58	31
S (F12)	26	50	24
S/G2 (F13)	10	35	55
G2/M (F16)	11	32	56

Table S2C. Percentage of Cells with Different Nanog Signal Intensity Levels, Related to Figure 3

	Low	Medium	High
G1 (F8)	31	49	19
G1 (F9)	30	54	16
G1/S (F10)	26	55	19
S (F11-12)	26	60	14
S/G2 (F13)	24	61	15
G2/M (F16)	28	61	12
