# Supplementary material for: DNA Synthesis Is Required for Reprogramming Mediated by Stem Cell Fusion
Source: Cell. 2013 Feb 14;152(4):873–83. doi: 10.1016/j.cell.2013.01.012 (PMC3605571; doi:10.1016/j.cell.2013.01.012)
Supplement: Document S2. Article plus Supplemental Information [file mmc2.pdf]

DNA Synthesis Is Required for Reprogramming Mediated by Stem Cell Fusion

Tomomi Tsubouchi,[1,2,3] Jorge Soza-Ried,[1,3] Karen Brown,[1] Francesco M. Piccolo,[1] Irene Cantone,[1] David Landeira,[1] Hakan Bagci,[1] Helfrid Hochegger,[2] Matthias Merkenschlager,[1] and Amanda G. Fisher[1,*]
[1]Lymphocyte Development Group, MRC Clinical Sciences Centre, Imperial College London, Du Cane Road, London W12 0NN, UK
[2]MRC Genome Damage and Stability Centre, Science Park Road, University of Sussex, Falmer, Brighton BN1 9RQ, UK
[3]These authors contributed equally to this work
*Correspondence: amanda.fisher@csc.mrc.ac.uk
http://dx.doi.org/10.1016/j.cell.2013.01.012

SUMMARY

Embryonic stem cells (ESCs) can instruct the conversion of differentiated cells toward pluripotency following cell-to-cell fusion by a mechanism that is rapid but poorly understood. Here, we used centrifugal elutriation to enrich for mouse ESCs at sequential stages of the cell cycle and showed that ESCs in S/G2 phases have an enhanced capacity to dominantly reprogram lymphocytes and fibroblasts in heterokaryon and hybrid assays. Reprogramming success was associated with an ability to induce precocious nucleotide incorporation within the somatic partner nuclei in heterokaryons. BrdU pulse-labeling experiments revealed that virtually all successfully reprogrammed somatic nuclei, identified on the basis of Oct4 re-expression, had undergone DNA synthesis within 24 hr of fusion with ESCs. This was essential for successful reprogramming because drugs that inhibited DNA polymerase activity effectively blocked pluripotent conversion. These data indicate that nucleotide incorporation is an early and critical event in the epigenetic reprogramming of somatic cells in experimental ESC-heterokaryons.

INTRODUCTION

Epigenetic reprogramming is a feature of normal embryonic development (Feng et al., 2010) that can also be induced experimentally using a range of strategies (Gurdon and Melton, 2008; Yamanaka and Blau, 2010). For example, differentiated somatic nuclei can regain pluripotency upon injection into oocytes (nuclear transfer) or through the forced expression of specific combination of transcription factors that induce a pluripotent stem (iPS) cell state (Gurdon, 1960; Takahashi and Yamanaka, 2006). Conversion of somatic cells toward pluripotency is associated with distinctive changes in the chromatin and DNA methylation status of the somatic genome (Deng et al., 2009; Simonsson and Gurdon, 2004) thought to be important for stable re-expression of core pluripotency factors such as Oct4, Sox2, and Nanog (reviewed by Papp and Plath, 2011). A third strategy for reprogramming somatic cells is by cell-cell fusion. There is an accumulating literature describing fusions between embryonic stem cells, embryonic carcinoma (EC) and embryonic germ (EG) cell lines with somatic cell partners such as thymocytes, lymphocytes, fibroblasts, or hepatocytes derived from the same or a different species (Miller and Ruddle, 1976; reviewed by Soza-Ried and Fisher, 2012). Collectively, these experiments have shown that somatic nuclei can be reprogrammed to acquire the epigenetic and developmental properties of their pluripotent partner (Ambrosi et al., 2007; Cowan et al., 2005; Do et al., 2007; Foshay et al., 2012; Matveeva et al., 1998; Pereira et al., 2008; Tada et al., 1997, 2001; Tat et al., 2011). Although the molecular mechanisms that determine the success and direction (or dominance) of this conversion are not fully understood, complete reprogramming is achieved 5–7 days after fusion with ESC, EG, and EC cells and is thought to occur in two steps. First, transient heterokaryons are formed in which both parental nuclei remain spatially discrete but share a common cytoplasm. Low levels of pluripotent gene expression from the somatic partner are initiated in a proportion of heterokaryons and increase over a 3–4 day period before the parental nuclei fuse to generate hybrids (Pereira et al., 2008). This second step has been proposed to stabilize or "fix" newly acquired gene expression profiles, enabling the resulting tetraploid cells to generate pluripotent colonies (reviewed by Serov et al., 2011). Because the first step occurs in the absence of cell division, it has been generally assumed that DNA synthesis is not required to initiate reprogramming.

Although some evidence supports this view (Bhutani et al., 2010), other studies have suggested that DNA synthesis may be required to reverse *cis*-mediated silencing of genes such as *Oct4* and *Nanog* (Foshay et al., 2012) or have suggested that somatic genome reprogramming occurs during the first cell cycle (Han et al., 2008). In this regard, classic cell fusion experiments performed more than 40 years ago using HeLa cells (Rao and Johnson, 1970) had shown that early (or precocious) DNA synthesis is induced in G1-phase cells upon fusion with

cells at later stages of the cell cycle (in S or G2 phases). As DNA synthesis provides an unrivaled opportunity for chromatin and nucleosome remodeling as well as changes to DNA methylation, it is important to establish whether there is any involvement of DNA synthesis in heterokaryon-mediated reprogramming in order to understand the mechanisms behind this conversion.

Embryonic stem cells and the pluripotent cells of the epiblast from which they arise, have a very unusual cell-cycle structure characterized by a short cell-cycle time, truncated G1 phase, and a large proportion of cells in DNA synthesis (S) phase (Fluckiger et al., 2006; White and Dalton, 2005). Pluripotent cells in the mouse epiblast devote more than 50% of cell-cycle time to S phase and a similarly high proportion of mouse ESC, EG, and EC cells (35%–50%) are reported to be in S phase (Savatier and Afanassieff, 2002; Stead et al., 2002). This unusual profile is associated with high levels of Cdk activity and anaphase-promoting complex/cyclosome (APC/C) substrates present throughout the cell cycle (Fujii-Yamamoto et al., 2005; Koledova et al., 2010b; Yang et al., 2011). A recent report has suggested that Cdk activity in ESCs may oscillate in a manner that is muted as compared with differentiated or somatic cells (Ballabeni et al., 2011). Although the biological consequences of this unusual cell cycle are not known, evidence that ESCs loose this profile upon differentiation (Bar-On et al., 2010; Calder et al., 2013; Koledova et al., 2010a; Orford and Scadden, 2008) and conversely, that somatic cells regain it when reprogrammed (Ghule et al., 2011; Ruiz et al., 2011; Singh and Dalton, 2009), have suggested that it may be important for rapid self-renewal of pluripotent cells.

One of the consequences of ESCs having impaired or muted cell-cycle checkpoints is that many of the drugs that have been traditionally used to synchronize or block somatic cells at specific stages of the cell cycle are often either ineffective or promote differentiation in ESCs, rendering cell-cycle studies in undifferentiated ESCs problematic (Calegari and Huttner, 2003; Han et al., 2008; Neganova and Lako, 2008; Ruiz et al., 2011). To circumvent this, we have optimized a biophysical cell separation method to enrich for ESCs in discrete phases of the cell cycle. Using this methodology we asked whether the ability of ESCs to dominantly reprogram differentiated cells was influenced by their cell-cycle stage. Our results show that ESCs in late S/G2 phases of the cell cycle have a markedly enhanced ability to reprogram somatic cells and provide evidence that this is because they induce the somatic nucleus to undergo a round of precocious DNA synthesis shortly after fusion.

RESULTS

Cell-Cycle Synchronization of ESCs by Centrifugal Elutriation

Counterflow centrifugal elutriation allows the separation of heterogeneous cell populations into fractions of uniform size and density (Banfalvi, 2008). As size and density reflect cell-cycle stage, we used this approach to isolate mouse ESCs at sequential stages of the cell cycle from undifferentiated cultures. Briefly, single cell suspensions of E14 ESCs were loaded into an elutriation chamber and centrifuged at constant speed. Fractions were collected at increasing flow rates (6–17 ml/min, F8 to F16) and evaluated for their DNA content by staining with propidium iodide (PI) followed by fluorescence-activated cell sorting (FACS) analysis. Typical DNA content profiles of unsynchronized ESCs and of sequential elutriated fractions are shown in Figure 1A (top and bottom panels, respectively) where the gates used to define G1, G2, and S phase are marked. Fractions F8 and F9 contained predominantly G1-phase ESCs (>80%), and fraction 16 was enriched for cells in G2 (>70%), whereas S-phase cells centered around fraction F12. The consistency of this separation approach was confirmed in five independent experiments (Figure S1A available online). To quantify cells undergoing DNA synthesis within each fraction we also subjected samples to a 45 min pulse of BrdU (100 μM) after elutriation and then identified and scored BrdU incorporating cells using immunofluorescence microscopy, as illustrated in Figure 1B (anti-BrdU, green). Among undifferentiated asynchronous ESC cultures BrdU label was routinely detected in $\sim$35% of cells and the pattern of BrdU distribution within nuclei was similar to the patterns previously reported for S-phase stages in somatic cells (Azuara et al., 2003; McNairn and Gilbert, 2003), as shown here for human B cells (hB) (Figure 1B and legend). In particular, early S phase (I, II) and late S phase (IV, V) patterns were detected in 42% and 20% of BrdU-labeled mouse ESCs and characteristically marked DNA replication at either diffuse euchromatic sites or within blocks of heterochromatin, respectively. Mid-S-phase cells (III) accounted for the remaining 38% of BrdU-labeled cells in which DNA synthesis was focused at either perinuclear domains (a), perinucleolar sites (b) or within dispersed sites of DAPI-intense heterochromatin (c). Using this BrdU pulse-labeling strategy we enumerated S-phase cells within elutriated ESC samples (Figure 1C). Fractions F11 to F12 were enriched for S-phase cells (>60% of cells incorporated BrdU, consistent with previous PI staining profiles). Fractions F8 and F9 contained relatively few S-phase cells (8%–14%) with labeling patterns indicative of early S phase (I and II). Fraction F16 typically contained 20%–30% BrdU positive cells and these cells showed a typical late S-phase distribution pattern (IV and V).

As drug-based treatments that arrest or delay ESC cell-cycle progression have been reported to promote differentiation and cell death (Burdon et al., 2002; Calegari and Huttner, 2003; Neganova and Lako, 2008; Orford and Scadden, 2008; Ruiz et al., 2011) we asked whether elutriated ESC samples successfully resumed cell cycle upon reculture. Fractions F8/F9, that were relatively homogeneous and enriched for G1-phase ESCs, were monitored for cell-cycle progression using PI staining at successive culture times (Figure 1D). G1-enriched ESC samples (F9, day 0) progressed through to S (day 1) and G2 (day 2) phases of the cell cycle, and subsequently showed a PI profile that resembled nonsynchronous ESC cultures (day 3). No increase in cell differentiation or significant loss of cell viability was detected in these cultures throughout the time course. Furthermore, a direct comparison of cell survival in nonsynchronous, G1-, S-, and G2/M-enriched ESCs cultured immediately after elutriation showed a similar viability between fractions (Figure S1B).

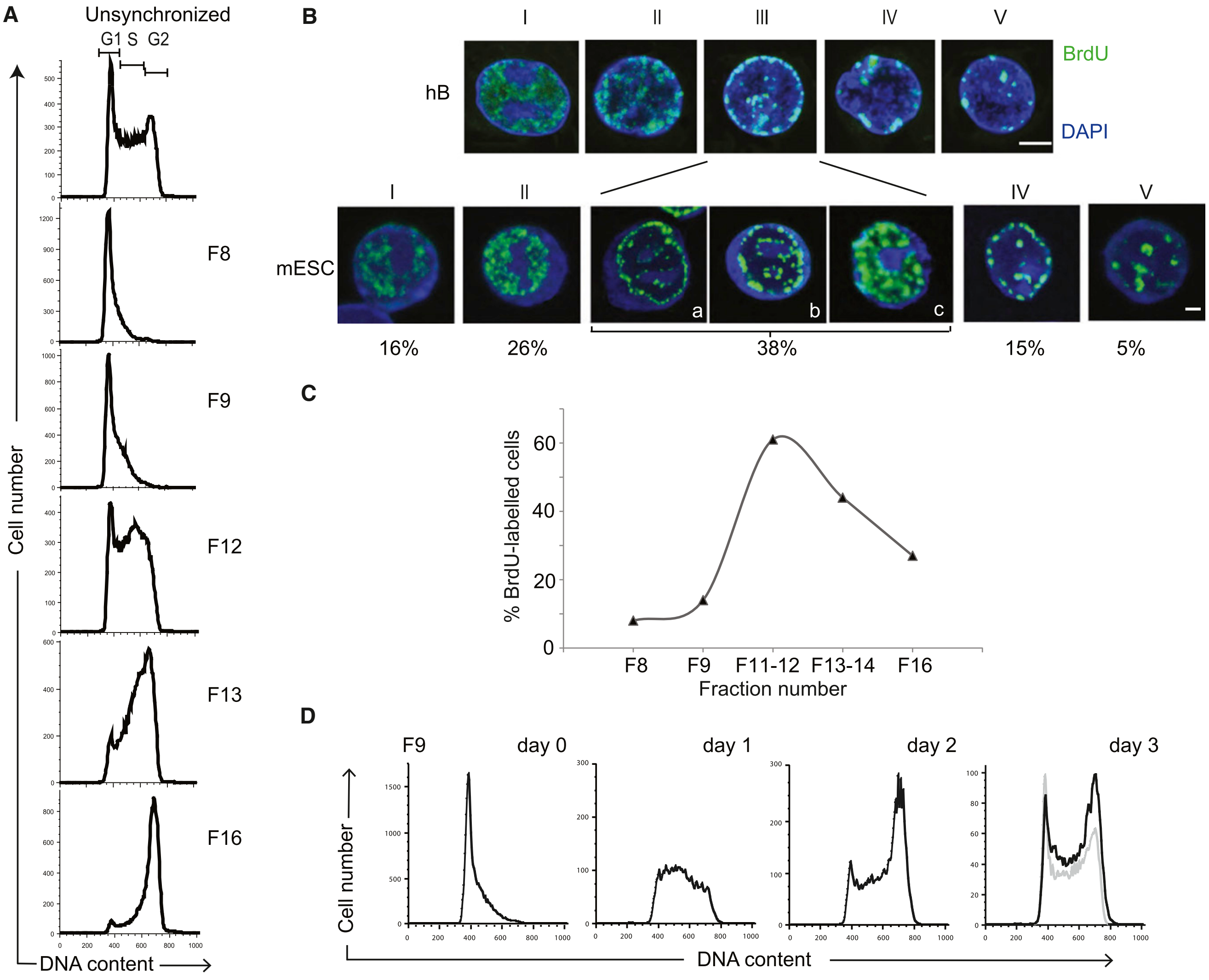

Figure 1. Separation of Mouse ESCs According to Cell-Cycle Stage Using Counterflow Centrifugal Elutriation

(A) Unsynchronized mouse E14tg2A ESCs cells were subjected to counterflow centrifugal elutriation and sequential fractions (F8, F9, F12, F13, F16, denoting flow rates) were stained with propidium iodide (PI) to assess DNA content by FACS. Gates used to define cells in G1, S, or G2/M are indicated in the top panel.

(B) BrdU labeling patterns that characterize successive stages of S phase in human B (hB) and mouse ESCs (mESC) are shown. Early S (I) phase is distinguished by a fine diffuse labeling of multiple euchromatic sites that gradually increase in number and intensity by stage II. Mid S phase (III) shows BrdU incorporation at the nuclear periphery (IIIa) and outlining nucleoli (IIIb), and in ESCs a pronounced increase in the overall number of foci (IIIc). In later stages (IV and V) large constitutive heterochromatin domains are evident. Scale bars, 5 μm.

(C) The abundance of S-phase cells in each elutriated fraction was determined by BrdU pulse labeling (45 min, 100 μM) and anti-BrdU immunostaining.

(D) Mouse ESCs enriched for G1 using counterflow centrifugal elutriation (F9) were cultured for 1–3 days to monitor progression through the cell cycle following elutriation. Unsynchronized ES cells are provided for comparison (in gray), and additional information is given in Figure S1.

ESC Reprogramming Capacity Varies with Cell-Cycle Stage

A previous study of hybrids generated between mouse thymocytes or fibroblasts fused with mouse ESCs that had been grown to different degrees of confluence (Sullivan et al., 2006) had suggested that reprogramming could be optimized using stem cells enriched for G2/M phases of the cell cycle. We used counterflow centrifugal elutriation to isolate cell-cycle stages from other variables that may occur in cultures grown to different densities. The ability of elutriated ESC samples to stably reprogram mouse B lymphocytes was compared using puromycin resistant mouse B cell targets that carried a silent *Oct4-GFP* transgene (GOF18ΔPE) (Yeom et al., 1996). These cells were fused in a 1:1 ratio with unsynchronized or elutriated mouse ESC fractions and the resulting cells were plated at limiting dilution in drug-containing media for 12 days as described previously (Pereira et al., 2010) (Figure 2A). Puromycin-resistant hybrid colonies expressing alkaline phosphatase (AP^+, $Puro^+$) were enumerated and compared to values obtained with non-synchronized ESCs (Figure 2B). In parallel, some colonies were

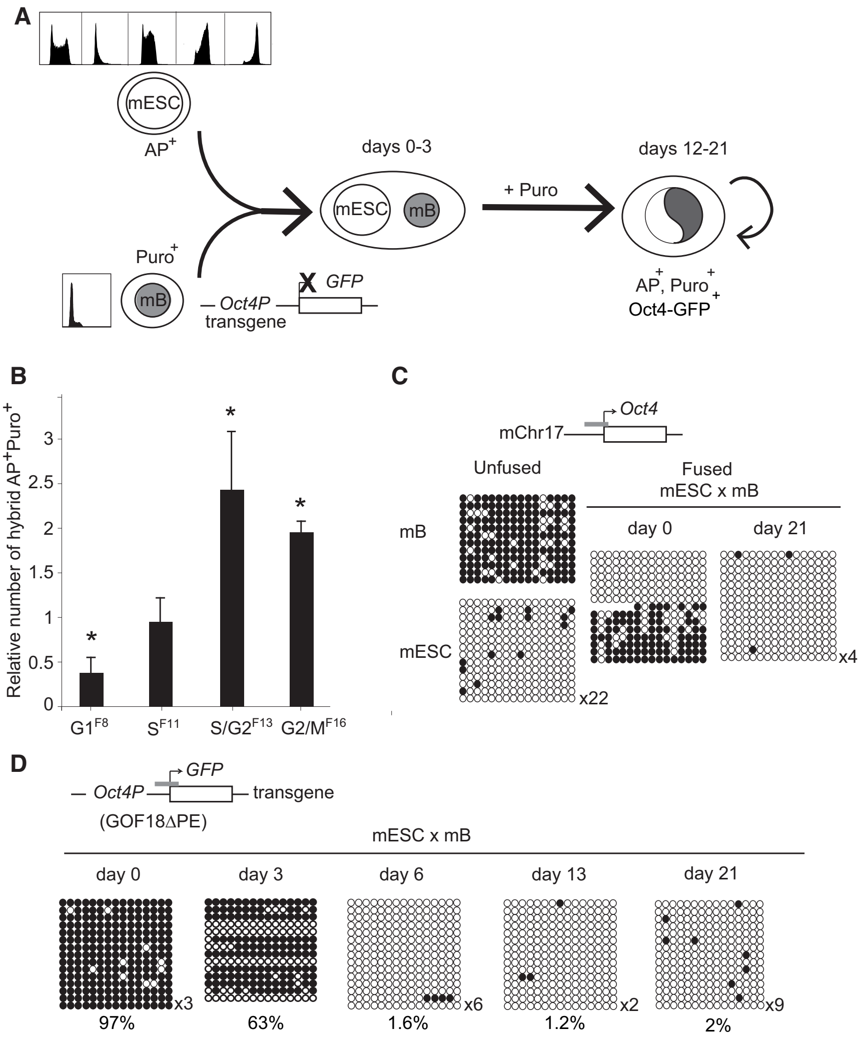

Figure 2. ESCs at Late Stages of the Cell Cycle Are More Efficient at Reprogramming B Cells Than Unsynchronized ESCs or G1-Phase Cells

(A) Strategy used for generating hybrids between mESCs (enriched in G1, S, S/G2, or G2/M) and puromycin-resistant ($Puro^+$) mouse B cells (mB) derived from an *Oct4-GFP* transgenic line (GOF18ΔPE) (Yeom et al., 1996). B cells were predominantly in G1 ($\geq$75%), as judged by PI staining. Successfully fused cells were plated in media supplemented with puromycin for 21 days. Immediately following cell fusion, the parental mESC and mB nuclei remained discrete within a single cell body supported by a shared cytoplasm for up to 3 days. Subsequently, the nuclei fuse giving rise to a proportion of stable proliferating hybrid cells resistant to puromycin and positive for alkaline phosphatase activity and Oct4 expression.

(B) Reprogramming efficiency of ESCs at different cell-cycle stages was evaluated by scoring the number of Puro-resistant hybrid colonies positive for AP activity (AP^+). Results show the number of AP^+ colonies expressed relative to colonies obtained with unsynchronized mESC (shown as 1), where error bars represent the SD of two to three independent experiments and asterisks denote statistical significance ($p < 0.05$, t test).

(C and D) DNA methylation at the endogenous (mCHr17) (C) and transgenic (GOF18ΔPE) (D) *Oct4* locus in hybrid cells was assessed by bisulfite sequence analysis, where closed circles represent methylated CpG and open circles show unmethylated CpG. In (D) the kinetics of loss of DNA methylation at the B cell derived transgenic *Oct4* locus was monitored using a previously described approach and primers (Han et al., 2008) and the percentage values indicate the proportion of CpG methylated sequences.

See also Figure S2 and Table S1.

expanded to evaluate Oct4-GFP expression, DNA content, karyotype, and the potential of the resulting hybrid cells to differentiate. This analysis indicated that S/G2-enriched fractions of mouse ESCs (F13) generated at least 5-fold more pluripotent hybrid colonies than G1-enriched fractions (F8) and supported the idea that ESCs at later stages of cell cycle have a more potent reprogramming capacity. This enhanced reprogramming (Figure 2B; Table S1) was not a reflection of intrinsic differences in the survival, cloning or fusion efficiency of S/G2, as shown in control experiments (Figures S2A and S2B). Importantly, hybrid clones generated by fusing mouse B cells with either unsynchronized or G2-enriched ESC re-expressed the Oct4-GFP transgene robustly (Figures S2C and S2D), were tetraploid (Figure S2D) and able to differentiate upon LIF withdrawal into mesoderm, endodermal, and ectodermal cell types (as exemplified by clones 1,6, and 3,10, respectively; Figures S2D–S2F). Consistent with full reprogramming of hybrid cells, a bisulfite analysis of DNA methylation showed a near complete loss of methylated CpG residues across endogenous mouse *Oct4* alleles by day 21 (Figure 2C). To estimate when after fusion this reprogramming-associated loss of DNA methylation had occurred, we next performed bisulfite analysis of the promoter region of the somatically derived *Oct4-GFP* transgene. This region is heavily methylated in the parental mouse B cells (97%, Day 0) but hypomethylated in reprogrammed hybrids (2% methylation, day 21). Time course experiments revealed that DNA demethylation of the reporter was evident as early as 3 days after fusion (Figure 2D), consistent with previous studies showing that Oct4 activation is an early event required for ESC fusion-mediated reprogramming (Han et al., 2008; Pereira et al., 2008).

Rapid and Potent Reprogramming of Human Somatic Cells Fused with S/G2-Enriched Mouse ESCs

To explore the possible mechanisms that underlie the improved reprogramming capacity of S/G2-enriched mouse ESCs we performed heterokaryon analyses using human B cells or human fibroblasts as targets (Figure 3A). This approach allows the earliest steps in reprogramming to be followed by employing a combination of species-specific antibodies, fluorescence in situ hybridization (FISH) probes, and RT-PCR primers to discriminate events that occur within individual mouse and human (somatic) nuclei after cellular fusion (Pereira et al., 2008;

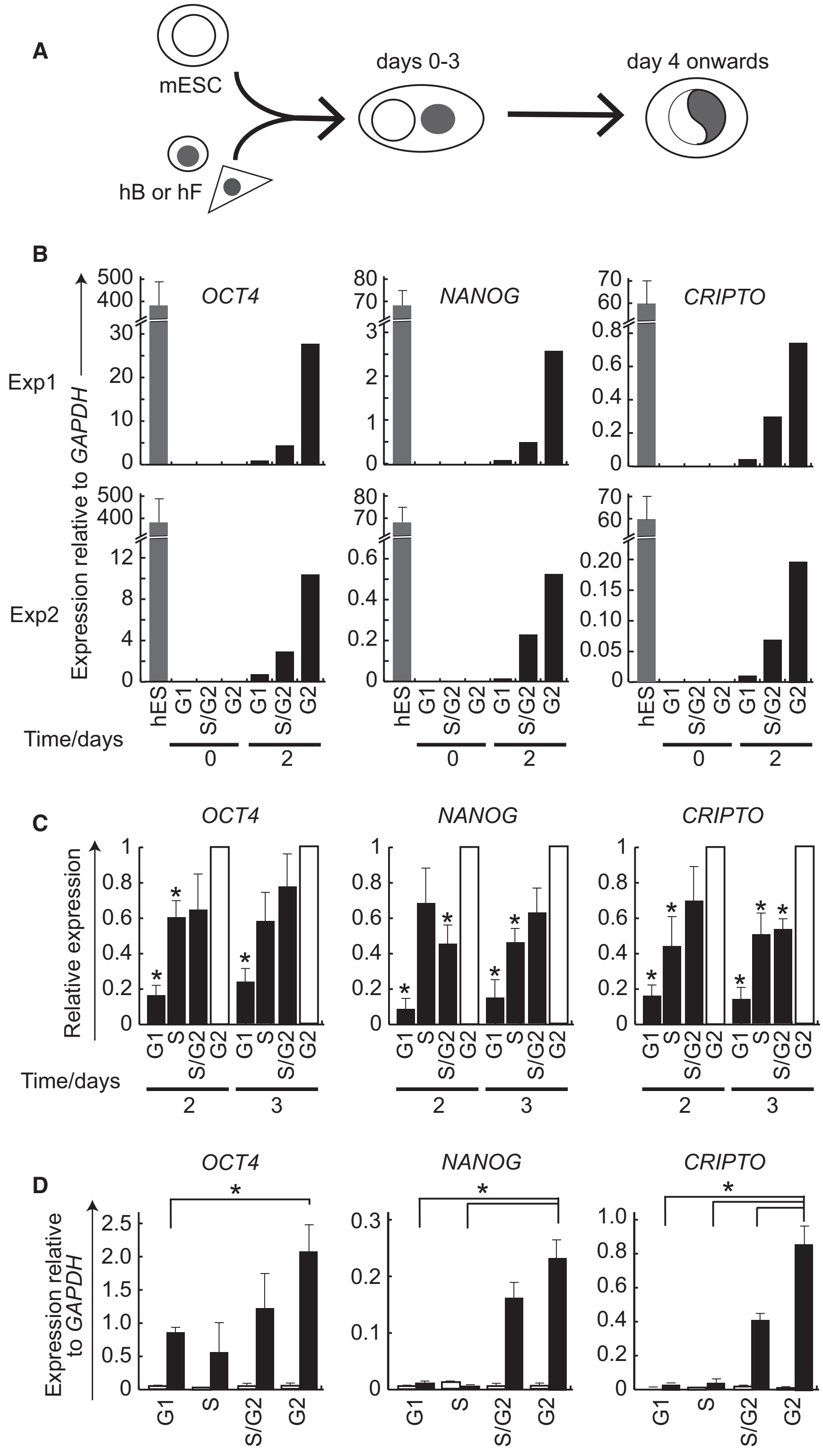

Figure 3. Mouse ESCs Enriched for S and G2 Phases of the Cell Cycle Efficiently Reprogram Human B Lymphocytes and Fibroblasts in Heterokaryons

(A) Experimental strategy for generating interspecies heterokaryons. Mouse ESCs (mESC) were fused in a 1:1 ratio with human B (hB) or human fibroblasts (hF) cells using PEG and cultured in ESC media supplemented with puromycin.

(B) mESCs enriched according to cell-cycle stage (G1 = F8/9, G1/S = F11/12, S/G2 = F13/14, and G2/M = F15/16) were fused with hB cells and hES-specific gene expression was assessed using RT-qPCR and species-specific primers. Gene expression was calculated relative to *GAPDH*, using the human ESC cell line NCL1 as a positive control (Pereira et al., 2008). Two independent experiments are shown as examples.

(C) The potency of mESCs to induce *OCT4*, *NANOG*, and *CRIPTO* expression from hB cells, 2 and 3 days after fusion was assessed in seven independent experiments. Values were normalized to G2-enriched samples, error bars denote SE from the mean and values statistically different from G2 ($p < 0.05$; single sample t test) are marked with an asterisk.

(D) In similar experiments using human fibroblasts as targets, human pluripotency gene induction was assessed (as above), and expression levels at day 0 (white bars) and day 4 (black bars) are shown. Error bars denote SE from the mean of three independent experiments, and asterisks indicate a significant difference with a p value (t test) of < 0.05.

See also Figure S3 and Table S2.

Piccolo et al., 2011). Human B cells were fused in a 1:1 ratio with unsynchronized or G1-, S-, G2-enriched mouse ESC fractions and expression of human pluripotency genes induced within these heterokaryons was compared using qPCR (Pereira and Fisher, 2009). Low levels of human *OCT4* (*POU5F1*), *NANOG*, *CRIPTO* (Figure 3B), *DNMT3b*, *REX1*, *FGFR1*, *FGF2*, and *TLE1* (Figure S3A) transcripts were detected 2 days after fusion with mouse ESCs (Pereira et al., 2010, 2008). The expression of human pluripotency genes was consistently higher in fusions performed with S/G2 ESCs as compared with either G1 or asynchronous cells (Figures 3B and S3B, top). This enhanced reprogramming capacity of late S/G2 and G2-enriched mouse ESC fractions was reproducible and statistically significant (Figure 3C). To determine whether somatic targets other than lymphocytes were also susceptible, we also fused elutriated mouse ESC fractions with human fibroblasts. As shown in Figures 3D and S3B (lower), reprogramming of fibroblasts was also significantly enhanced following fusion with S/G2- and G2-enriched mESCs, as indicated by increased induction of human *OCT4*, *NANOG*, and *CRIPTO* transcripts.

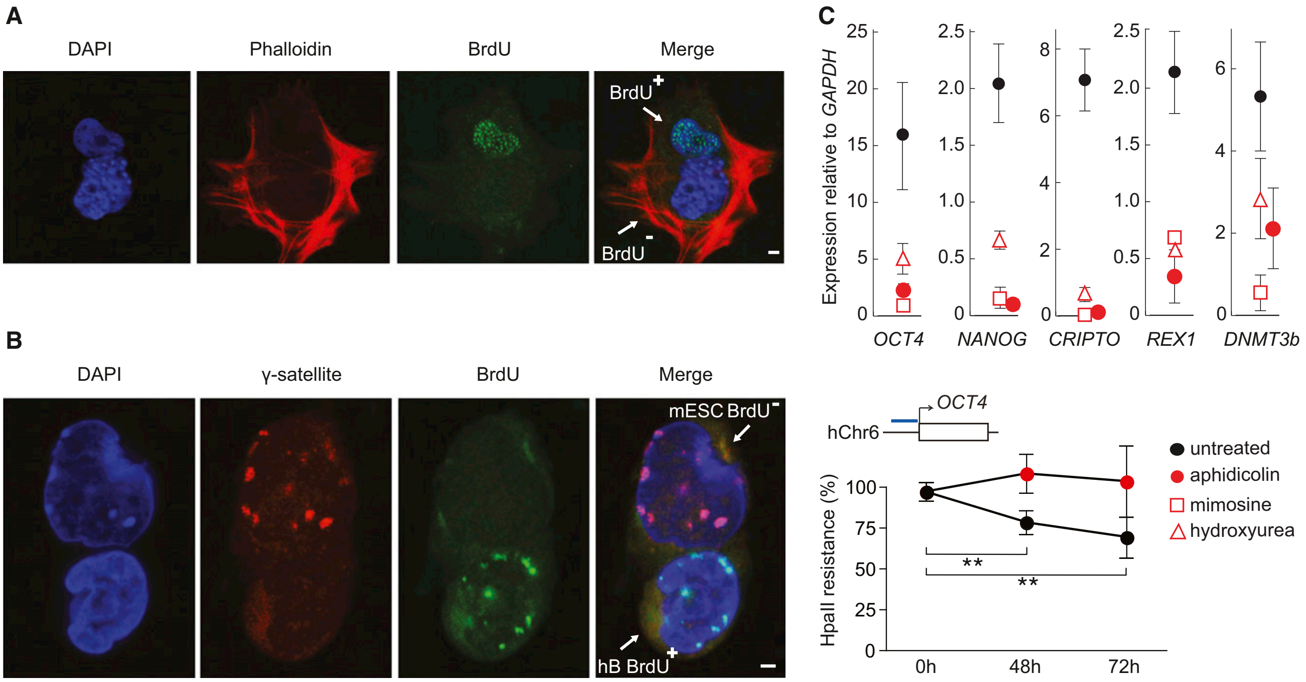

Figure 4. Human Somatic Nuclei Undergo Precocious DNA Synthesis in Heterokaryons Formed with Mouse ESCs, and This Is Required for Successful Reprogramming

(A) Confocal image of a representative heterokaryon (hB x mESC) at day 1, that was labeled for 45 min with BrdU and then stained to reveal incorporated BrdU (green), DAPI (blue), and Phalloidin (red).

(B) Confocal Image showing simultaneous FISH detection of γ-satellite probe and BrdU labeling of hB × mESC heterokaryons at day 1, where γ-satellite (red) selectively labels mouse nuclei and BrdU incorporation (green) by hB nuclei is evident. The extent of BrdU incorporation by hB nuclei in heterokaryons formed with unsynchronized, S/G2-enriched or G1-enriched mESCs, was compared using these approaches and the results are shown in Table 1. DAPI nuclear staining is shown in blue. Scale bars, 2 μm.

(C) Reprogramming of hB by mESCs was assayed 48 hr after heterokaryon formation in the absence (black) or presence (red) of 200 μM hydroxyurea (open triangle), 300 μM mimosine (open square), or 2 μg/ml aphidicolin (closed circle). Values are the mean and SE of five independent experiments. Differences between treated and untreated samples were significant with all drugs for *OCT4*, *NANOG*, and *CRIPTO*, and only with mimosine for *DMNT3b* (p value < 0.05, t test). DNA methylation at the *OCT4* promoter was assessed 48 hr and 72 hr after fusion in the absence (black) or presence (red) of aphidicolin by HpaII digestion, where HpaII resistant fragments were quantified by qPCR using species-specific primers (blue bar indicate position), and normalized to undigested samples. Values shown are the mean and SD of four to six replicates in which the statistical significance is indicated by asterisks (p value < 0.005, t test).

See also Figure S4.

To understand the basis of this improved reprogramming capacity we initially examined the possibility that factors that are known to potentiate or inhibit iPS-based reprogramming (Yamanaka and Blau, 2010) might fluctuate during ESC cell cycle. We were, however, unable to detect any significant changes in the levels of mouse *Oct4*, *Klf4*, *Sox2*, *c-Myc*, *Nanog*, *p53*, or *p21* transcripts in cell-cycle-enriched mouse ESCs (Figure S3C) and protein levels remained unchanged for most candidates (Figure S3D). Although western blotting and immunofluorescence analysis showed slight increases in Oct4 and Sox2 levels upon cell-cycle progression (Figures S3D and S3E; Tables S2A and S2B), careful comparison of unsynchronized and G2-enriched samples indicated broadly comparable levels in both (Figure S3D, red box). We did not see any experimental evidence that these factors were either prematurely dissociated from the chromatin or exported to the cytoplasm ahead of mitosis (Figure S3F). Likewise, Nanog expression, which displays a characteristic periodicity in ESC cultures (Chambers et al., 2007) and has been shown to enhance experimental reprogramming (Silva et al., 2006; Theunissen et al., 2011), was independent on ESC cell-cycle stage (Table S2C; Figures S3C and S3D).

S/G2 ESCs Induce Precocious Nucleotide Incorporation in Somatic Nuclei Shortly after Fusion

An alternative explanation for the superior efficiency of S/G2-enriched ESCs to reprogram somatic cells is that S- or G2-phase cells were capable of inducing premature DNA synthesis and chromosome condensation in G1-phase targets (Johnson and Rao, 1970; Rao and Johnson, 1970) and that this may facilitate chromatin remodeling, DNA demethylation, and activation of critical genes, as has been previously suggested (De Carvalho et al., 2010; Mikkelsen et al., 2008). To examine this possibility, we fused human B and mouse ESCs in a 1:1 ratio and after 24 hr applied a pulse of BrdU (45 min, 100 μM) to enumerate nuclei in these cultures that were undergoing DNA synthesis. Heterokaryons containing BrdU-labeled nuclei were then visualized by DAPI (blue), phalloidin (red), and anti-BrdU (green) costaining as shown in Figure 4A. Most heterokaryons contained

Table 1. Incorporation of BrdU by Human B Nuclei Contained within 1 Day Heterokaryons Formed with Mouse ESCs

	$BrdU^+$ hB	Sample Size	%
Experiment 1			
hB × unsynchronized mESC	8	24	33
hB × S/G2-enriched mESC	30	35	86
Experiment 2			
hB × unsynchronized mESC	11	23	48
hB × S/G2-enriched mESC	14	19	74
Experiment 3			
hB × unsynchronized mESC	10	30	33
hB × S/G2-enriched mESC	27	35	77
hB × G1-enriched mESC	0	80	<1.3

Heterokaryons between human B (hB) and mouse ESC (1:1) were cultured for 1 day and pulse labeled with BrdU (100 μM/45 min), and resulting nucleotide incorporation in hB ($BrdU^+$hB) was assessed as shown in Figure 4. Heterokaryons formed with S/G2-enriched mESCs contained a higher proportion of hB nuclei that incorporated BrdU than heterokaryons generated with unsynchronized mESCs (p value < 0.003).

BrdU-labeled nuclei (>85%) 1 day after fusion suggesting that nucleotide incorporation was relatively common in these heterokaryons. The amount (intensity) and distribution of BrdU labeling was similar to that seen in somatic and ESCs during S phase. To discriminate human nuclei that were incorporating BrdU ($BrdU^+$ hB, green), we used gamma satellite probe (γ-satellite, red) to selectively mark mouse nuclei (Figure 4B). Using this approach, we scored $BrdU^+$ hB in heterokaryons 24 hr after fusing human B cells with either unsynchronized or G2-enriched ESCs. Data from three independent experiments showed that a large proportion of heterokaryons formed with S/G2-enriched ESCs contained hB nuclei that incorporated BrdU (74%–86%, Table 1). In heterokaryons generated with unsynchronized mouse ESCs this proportion was lower (33%–48%). By comparison, heterokaryons formed with G1-enriched ESCs lacked widespread BrdU incorporation and failed to induce DNA synthesis within most human B cell targets (0/80, Table 1). These data show that ESCs in S/G2 phase of cell cycle induce precocious nucleotide incorporation within their somatic partners during early stages of heterokaryon formation. Consistent with this observation, ESC multikaryons that contained multiple human B cell nuclei (as illustrated in Figure S4A) usually showed coordinated BrdU labeling patterns in all somatic nuclei. BrdU incorporation by somatic nuclei in heterokaryons could be detected as soon as 5–6 hr after fusion with a pattern that was typical of early S phase (Figure S4B). Importantly, BrdU labeling was not seen in most hB homokaryons (Figure S4C) or within human B cells that had been engulfed by mouse ESCs but had retained a discrete cell wall (Figure S4D). This suggests that precocious DNA synthesis in lymphocyte nuclei is an early feature induced by ESC that requires the establishment of a shared cytoplasm.

To determine whether nucleotide incorporation by somatic nuclei was likely to reflect widespread DNA repair or genuine DNA replication, we performed DNA FISH analysis and scored doublet FISH signals 24 hr after fusion. Using genomic probes for human α-globin and β-globin (loci that replicate early and late in B cells, respectively) (Brown et al., 2001) together with EdU (to visualize nucleotide incorporation), we routinely detected doublet signals for α-globin in human B nuclei 24 hr after fusion (24/25 nuclei, 98%), whereas β-globin appeared as singlet signals (exemplified in the mid-S-phase nucleus shown in Figure S4E). As doublets reflect the separation of newly replicated sister chromatids during S phase (Azuara et al., 2003; Selig et al., 1992) these data are consistent with human somatic nuclei undergoing DNA replication. Parallel experiments using human fibroblasts (IMR90, hF) showed widespread BrdU incorporation by somatic nuclei a day after fusion with unsynchronized ESCs (Figure S4F), with doublet FISH signals (*HUWE1*) clearly evident at days 2–3 (Figure S4G). These data confirmed human DNA replication in ESC-heterokaryons ahead of nuclear fusion.

DNA Replication Is Critical for Initiating Successful Reprogramming of Somatic Nuclei toward Pluripotency in ESC-Derived Heterokaryons

To evaluate whether DNA synthesis was required to initiate pluripotent gene expression from somatic nuclei, we fused human B cells and mouse ESCs and then monitored human pluripotent gene induction following treatment with agents that block DNA synthesis. Treatment of heterokaryons with aphidicolin (a drug that inhibits DNA polymerase activity), mimosine (that arrests cells in late G1) or with hydroxyurea (to prevent late origin firing) for 48 hr severely compromised the induction of a panel of human pluripotency genes as compared with untreated controls (Figure 4C, top). DNA demethylation at endogenous human *OCT4* alleles was evident in untreated cultures, as indicated by increased sensitivity to HpaII digestion (Figure 4C, bottom, black). However, in parallel cultures in which DNA polymerase activity was inhibited by aphidicolin treatment, we detected no corresponding change in sensitivity of the *OCT4* locus (Figure 4C, bottom, red). Taken together, these data suggested that precocious DNA synthesis within somatic nuclei was critical for remodeling and demethylating the human *OCT4* locus prior to its reactivation in heterokaryons.

Oct4 Re-Expression and Precocious DNA Synthesis

The incidence of precocious DNA synthesis among successfully reprogrammed ESC heterokaryons was evaluated in BrdU tracing experiments, using *Oct4-GFP* as a reporter. Briefly, mouse B cells carrying *Oct4-GFP* were fused with nonsynchronized ESCs and pulse labeled with BrdU (100 μM, 45 min) at different times early after fusion (at 6, 18, or 24 hr, or double-pulsed at 6 and 18 hr), washed and returned to culture. Two to 3 days after fusion heterokaryons that contained "reprogrammed lymphocytes" were identified on the basis of GFP re-expression and then examined to ask whether these cells retained BrdU that had been acquired during the time window of the pulse. A schematic representation of this experiment is depicted in Figure 5A. As shown in Figure 5B and illustrated in Figure 5C, most Oct4-GFP positive heterokaryons (green) identified 2-3 days after fusion had incorporated BrdU (red) within the first few hours of fusion. Specifically, we demonstrated that about a third of all successfully reprogrammed cells were marked by BrdU applied 6 hr postfusion and about two thirds of all successfully reprogrammed cells were marked by BrdU

A

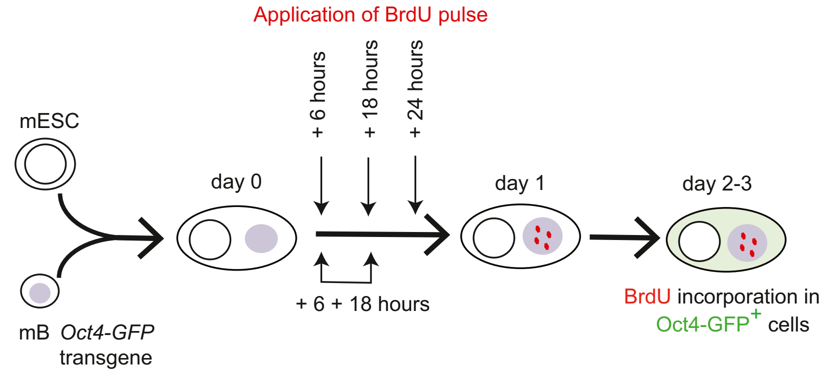

B

Time of BrdU pulse	BrdU labelled cells among Oct4–GFP$^+$ cells at 2-3 day	Sample size
+6 hours	33%, 37%	50
+18 hours	62%	46
+24 hours	76%	29
+6 +18 hours	90%	61

C

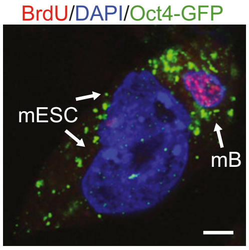

Figure 5. Somatic Nuclei that Re-Express Oct4 Show Widespread Nucleotide Incorporation within 24 hr of Heterokaryon Formation with ESCs

(A) Experimental strategy used to assess the kinetics of BrdU incorporation in successfully reprogrammed mB cells. Heterokaryons formed between mouse ESCs and B cells were pulse labeled (45 min) with BrdU at different times points after fusion (6, 18, or 24 hr) in separate experiments or pulse labeled twice at 6 and 18 hr after fusion in a single experiment. BrdU incorporation was analyzed among successful reprogrammed mouse B cells identified on the basis of Oct4-GFP re-expression at 2–3 days after fusion.

(B) Reprogrammed cells (33%–37%) had already incorporated BrdU during a pulse labeling applied 6 hr after fusion, a percentage that increased to 62% and 76% when fused cells were pulse labeled at 18 and 24 hr, respectively. Sequential pulse labeling at 6 and 18 hr demonstrates that the majority of successfully reprogrammed heterokaryons (90%) incorporated BrdU within a day of fusion.

(C) Confocal image of a successfully reprogrammed mouse B cell (mB, arrow) identified on the basis of Oct4-GFP (green) re-expression at 48 hr, that had incorporated BrdU (red) during a pulse applied 6 hr after PEG-mediated fusion with mESCs (mESC, arrow). Scale bar, 5 μm.

applied 18 hr postfusion. By applying a double pulse of BrdU at 6 and 18 hr after fusion, virtually all (90%) successfully reprogrammed B cells identified on the basis of Oct4 re-expression, were shown to have undergone DNA synthesis within a day of fusion with ESCs.

DISCUSSION

Here we provide quantitative evidence that dominant reprogramming of somatic cells by ESCs is enhanced using S/G2-enriched samples. This extends claims made from hybrid studies (Sullivan et al., 2006) showing that the potency of S/G2 ESCs occurs in heterokaryons when pluripotent gene expression is initiated. We found no evidence that the improved reprogramming efficiency of S/G2 ESCs was due to reprogramming factors present in cells at later stages of the cell cycle or their "release" into the cytoplasm prior to mitotic chromosome condensation. Rather, our data indicate that ESCs in S/G2 induce B cells or fibroblasts to undergo DNA synthesis within a day of fusion and heterokaryon formation. This is important for reprogramming as inhibitors of DNA synthesis such as hydroxyurea, mimosine and aphidicolin, blocked pluripotent gene induction in heterokaryons. We also found that virtually all heterokaryons that re-expressed a somatically derived *Oct4-GFP* transgene showed evidence of premature DNA synthesis occurring within the first day after fusion. Collectively, this indicates that early DNA synthesis is a critical feature of successful stem cell fusion-mediated reprogramming that has not been fully appreciated until now.

Interestingly, reprogramming studies using nuclear transfer have indicated that cell-cycle synchronization between the donor nucleus and recipient cytoplasm appears to be important (Campbell and Alberio, 2003; Campbell et al., 1996) and the ability of mammalian embryonic cytoplasm to support reprogramming has been shown to fluctuate with the cell cycle (Egli et al., 2007). Although synchronization of the cell cycle is not sufficient to determine successful reprogramming in heterokaryons per se (i.e., G1-phase ESCs do not efficiently reprogram G1-phase B cells), our data may highlight the importance of cell-cycle "compatibility" between nuclei inducing and nuclei undergoing reprogramming. Consistent with classical studies of cell-cycle progression (Blow and Laskey, 1988) and the cell fusion experiments reported by Johnson and Rao (1970) and Rao and Johnson (1970), we showed that fusing S/G2-phase ESCs with somatic cells induced precocious DNA synthesis in somatic nuclei. As 70%–75% of cultured human B cells are in G1, most of these targets would be expected to be already licensed for DNA replication (reviewed in Blow and Dutta, 2005) and therefore susceptible to S-phase promotion by S/G2 ESCs. In this regard, recent experiments using *Xenopus* egg extracts at the metaphase (M phase) stage have also shown that M phase can both drive DNA synthesis and improve reprogramming efficiency in nuclear transfer and in iPS assays (Ganier et al., 2011). iPS-based studies have, in addition, indicated that reprogramming can be accelerated by DNA synthesis and cell division (Hanna et al., 2009) as well as by agents that inhibit histone deacetylation (Huangfu et al., 2008) or interfere with the maintenance of DNA methylation (De Carvalho et al., 2010; Feng et al., 2009; Mikkelsen et al., 2008).

The demonstration that nucleotide incorporation is widespread among somatic nuclei in heterokaryons raises the possibility that DNA demethylation of genes that are critical

for reprogramming, such as *Oct4* (Simonsson and Gurdon, 2004) could be achieved by replication-dependent (passive) means. Previous studies have implicated AID-mediated deamination in active DNA demethylation in heterokaryons (Bhutani et al., 2010), and Gadd45 (growth arrest and DNA damage 45 protein) in active DNA demethylation during differentiation and stress response (Niehrs and Schäfer, 2012). In preimplantation embryos, both active and passive mechanisms have been implicated in genome-wide DNA demethylation in which Tet3-mediated conversion of 5-methylcytosine to 5-hydroxy-methylcytosine appears to be central (Gu et al., 2011; Inoue and Zhang, 2011). In ESCs, Tet1 and Tet2 family members are actively expressed (Ficz et al., 2011; Wu and Zhang, 2011; Xu et al., 2011) and recent studies have implicated Tet2 and Parp1 in iPS-based reprogramming (Doege et al., 2012). In an accompanying manuscript (Piccolo et al., 2013) we show that Tet1 and Tet2 participate in ESC- and EG-mediated heterokaryon reprogramming and are required to reset DNA methylation within the somatic genome. Interestingly, as conversion of 5-methylcytosine to 5-hydroxy-methylcytosine can occur in the presence of drugs that inhibit DNA replication (Piccolo et al., 2013), it seems likely that both active (DNA replication-independent) and passive (DNA replication-dependent) mechanisms may contribute to pluripotent reprogramming in ESC heterokaryons. This duality may be helpful in reconciling some of the conflicting data arising from reprogramming studies in vivo, as well as providing an explanation for the recent proposal that heterokaryon-mediated reprogramming is mechanistically biphasic (Foshay et al., 2012).

The observation that early DNA synthesis is prevalent in somatic nuclei fused with ESCs is however at odds with a previous report in which BrdU incorporation was not seen in heterokaryons formed between human fibroblasts and mouse ESCs (Bhutani et al., 2010). Although we do not at present have an explanation for this, it is unlikely that this discrepancy is due to the use of different somatic cells or to interspecies incompatibilities as we observed extensive BrdU incorporation using both human and mouse fibroblasts. In our hands AID expression (a putative mediator of 5-methylcytosine deamination) was not detectable in fused or unfused cells, as has been reported by others (Foshay et al., 2012). Regardless of the explanation for these differences, our study offers a fresh perspective on how reprogramming works as well as providing a simple and reliable method for enriching ESCs at different cell-cycle stages. This will be important for future reprogramming studies and to better understand the importance of the unusual cell-cycle structure in pluripotent stem cell self-renewal.

EXPERIMENTAL PROCEDURES

Cell Culture

E14Tg2a *Hprt*$^{-/-}$ (E14) mouse ESCs, EBV-transformed human B cells, and Abelson-transformed mouse B cells were cultured as described previously (Pereira et al., 2008).

Quantitative RT-PCR Analysis

RNA extraction and RT-qPCR was carried out as described previously (Pereira et al., 2008; primer sequences shown in Table S1).

Centrifugal Elutriation

Counter-flow centrifugal elutriation was carried out using JE-5.0 elutriator system (Beckman Coulter) in combination with MasterFlex peristaltic pump (Cole-Parmer Instrument) as described by Banfalvi (2008) and detailed in Extended Experimental Procedures.

Heterokaryons and Hybrid Assays

Heterokaryons were generated by fusing mouse ESCs enriched at different cell-cycle stages and human B cells (mESC/hB ratio 1:1) using polyethylene glycol (PEG) as described previously (Pereira and Fisher, 2009). Nonfused mouse ESCs were eliminated by supplementing the medium with HAT (20 μM hypoxanthine, 0.08 μM aminopterin, and 3.2 μM thymidine; Sigma) or Puromycin (1 μg/ml; Sigma). Nonfused hB cells were, where appropriate, eliminated using ouabain (10^{-5} M; Sigma) applied at least 6–18 hr after cell fusion, and details of immunofluorescence analyses are provided in Extended Experimental Procedures.

Hybrids between mESCs (enriched either in G1, S, S/G2, or G2/M) and puromycin-resistant (Puro^{+}) mB cells were generated by PEG-mediated fusion (mESC/mB ratio 1:1) (Pereira and Fisher, 2009). Nonfused mESCs were eliminated by the addition of Puromycin (1 μg/ml; Sigma), and alkaline phosphatase staining was performed on colonies 12 days after fusion.

Protein Extraction and Western Blot Analysis

Whole-cell protein extracts were prepared with Laemmli buffer, and nuclear and cytoplasmic protein extracts were prepared as described in Extended Experimental Procedures. Western blot analysis were performed using the following antibodies: goat polyclonal to Oct4 (1:2,000; sc-8628, Santa Cruz Biotechnology), Sox2 (1:2,000; sc-17320, Santa Cruz Biotechnology), and LaminB (1:5,000; sc-6216, Santa Cruz Biotechnology); Mouse monoclonal to c-Myc (9E10) (1:2000; sc-40, Santa Cruz Biotechnology) and Tubulin (1:2,000; T9026 Sigma-Aldrich); rabbit polyclonal to CTCF (1:500; ab70303 Abcam). The secondary antibodies used were anti-mouse HRP (1:2000, GE Healthcare), anti-rabbit HRP (1:5,000, GE Healthcare), and anti-goat HRP (1:2000, sc-2020 Santa Cruz Biotechnology). The Amersham ECL plus kit (GE Healthcare) was used for detection.

Immunofluorescence, FISH, and BrdU detection

Immunofluorescence analysis was performed (Terranova et al., 2006) using goat polyclonal to Oct4 (1:200, sc-8628, Santa Cruz Biotechnology) and Sox2 (1:200, sc-17320, Santa Cruz Biotechnology), and secondary antibodies at 1:400 dilution (Molecular Probes). BrdU incorporation and detection was performed in low light conditions as described by Azuara et al. (2003) where heterokaryons were grown on gelatinized coverslips for 1 day, pulse labeled with 100 μM BrdU added to the media for 45 min, and fixed with 2% paraformaldehyde in PBS for 20 min at room temperature. Blocking and washing was performed as described by Terranova et al. (2006). Phalloidin-Alexa Fluor 568 (Invitrogen A12380, diluted 1:50 in blocking buffer) and/or BrdU-FITC (Becton-Dickinson 347583, diluted 1:4 in blocking buffer) were applied for 30 min before final washes and mounting in Vector-shield containing 0.5 mg/ml DAPI (Sigma D9542). Three-dimensional DNA FISH for human α- and β-globin were performed as described (Brown et al., 2001) on heterokaryons growing on 0.2% gelatin-coated coverslips. To codetect EdU incorporated as a 45 min pulse label before cell fixation, the Click-iT kit (Invitrogen) was used according to manufacturer's instructions following the sodium borohydride step. Coverslips were subsequently washed with PBS, blocked as described in the 3D FISH protocol before postfixation and DNA denaturation, and kept in the dark following detection of EdU.

Bisulfite Sequencing Analysis

Bisulfite treatment of genomic DNA was performed with the EZDNA methylation kit (Zymogenetics). Bisulfite-converted DNA was used as template to amplify endogenous (*Oct4*) and transgenic *Oct4* (GOF18ΔPE) (t*Oct4*) (primers are available in Extended Experimental Procedures). PCR products were cloned into pCR2 vector (Invitrogen) and randomly sequenced.

HpaII Resistance Assay

Genomic DNA (5 μg/sample) was digested with HpaII (50 U, NEB), with MspI (100 U, NEB), or left untreated (undigested) for 4 hr at 37°C, followed by

proteinase K treatment (30 min at 40°C). HpaII-resistance was quantified using qPCR and primers that flanked an HpaII/MspI-sensitive site in the promoter region of *OCT4*. Ct values were normalized to a region lacking an HpaII/MspI site and to untreated controls. HpaII resistance was calculated as the percentage difference between HpaII (test) and MspI (total) digested samples. Primers are available in Extended Experimental Procedures.

SUPPLEMENTAL INFORMATION

Supplemental Information includes Extended Experimental Procedures, four figures, and two tables and can be found with this article online at http://dx.doi.org/10.1016/j.cell.2013.01.012.

ACKNOWLEDGMENTS

We thank C.F. Pereira, H. Tsubouchi, and E. Petretto for help and discussions; S. Amoils for GG1 and ME2.5 probes for DNA FISH; and T. Carr and members of the CSC FACS facility for advice and technical support. This work was funded by the Medical Research Council UK (F.M.P., K.B., M.M., A.G.F.), the Human Frontier Science Program (T.T. and I.C.), and the European Research Council (A.G.F. and J.S.-R).

Received: October 1, 2011
Revised: February 14, 2012
Accepted: January 7, 2013
Published: February 14, 2013

REFERENCES

Ambrosi, D.J., Tanasijevic, B., Kaur, A., Obergfell, C., O'Neill, R.J., Krueger, W., and Rasmussen, T.P. (2007). Genome-wide reprogramming in hybrids of somatic cells and embryonic stem cells. Stem Cells *25*, 1104–1113.

Azuara, V., Brown, K.E., Williams, R.R., Webb, N., Dillon, N., Festenstein, R., Buckle, V., Merkenschlager, M., and Fisher, A.G. (2003). Heritable gene silencing in lymphocytes delays chromatid resolution without affecting the timing of DNA replication. Nat. Cell Biol. *5*, 668–674.

Ballabeni, A., Park, I.H., Zhao, R., Wang, W., Lerou, P.H., Daley, G.Q., and Kirschner, M.W. (2011). Cell cycle adaptations of embryonic stem cells. Proc. Natl. Acad. Sci. USA *108*, 19252–19257.

Banfalvi, G. (2008). Cell cycle synchronization of animal cells and nuclei by centrifugal elutriation. Nat. Protoc. *3*, 663–673.

Bar-On, O., Shapira, M., Skorecki, K., Hershko, A., and Hershko, D.D. (2010). Regulation of APC/C (Cdh1) ubiquitin ligase in differentiation of human embryonic stem cells. Cell Cycle *9*, 1986–1989.

Bhutani, N., Brady, J.J., Damian, M., Sacco, A., Corbel, S.Y., and Blau, H.M. (2010). Reprogramming towards pluripotency requires AID-dependent DNA demethylation. Nature *463*, 1042–1047.

Blow, J.J., and Laskey, R.A. (1988). A role for the nuclear envelope in controlling DNA replication within the cell cycle. Nature *332*, 546–548.

Blow, J.J., and Dutta, A. (2005). Preventing re-replication of chromosomal DNA. Nat. Rev. Mol. Cell Biol. *6*, 476–486.

Brown, K.E., Amoils, S., Horn, J.M., Buckle, V.J., Higgs, D.R., Merkenschlager, M., and Fisher, A.G. (2001). Expression of alpha- and beta-globin genes occurs within different nuclear domains in haemopoietic cells. Nat. Cell Biol. *3*, 602–606.

Burdon, T., Smith, A., and Savatier, P. (2002). Signalling, cell cycle and pluripotency in embryonic stem cells. Trends Cell Biol. *12*, 432–438.

Calder, A., Roth-Albin, I., Bhatia, S., Pilquil, C., Lee, J.H., Bhatia, M., Levadoux-Martin, M., McNicol, J., Russell, J., Collins, T., et al. (2013). Lengthened G1 phase indicates differentiation status in human embryonic stem cells. Stem Cells Dev. *22*, 279–295.

Calegari, F., and Huttner, W.B. (2003). An inhibition of cyclin-dependent kinases that lengthens, but does not arrest, neuroepithelial cell cycle induces premature neurogenesis. J. Cell Sci. *116*, 4947–4955.

Campbell, K.H., and Alberio, R. (2003). Reprogramming the genome: role of the cell cycle. Reprod. Suppl. *61*, 477–494.

Campbell, K.H., Loi, P., Otaegui, P.J., and Wilmut, I. (1996). Cell cycle co-ordination in embryo cloning by nuclear transfer. Rev. Reprod. *1*, 40–46.

Chambers, I., Silva, J., Colby, D., Nichols, J., Nijmeijer, B., Robertson, M., Vrana, J., Jones, K., Grotewold, L., and Smith, A. (2007). Nanog safeguards pluripotency and mediates germline development. Nature *450*, 1230–1234.

Cowan, C.A., Atienza, J., Melton, D.A., and Eggan, K. (2005). Nuclear reprogramming of somatic cells after fusion with human embryonic stem cells. Science *309*, 1369–1373.

De Carvalho, D.D., You, J.S., and Jones, P.A. (2010). DNA methylation and cellular reprogramming. Trends Cell Biol. *20*, 609–617.

Deng, J., Shoemaker, R., Xie, B., Gore, A., LeProust, E.M., Antosiewicz-Bourget, J., Egli, D., Maherali, N., Park, I.H., Yu, J., et al. (2009). Targeted bisulfite sequencing reveals changes in DNA methylation associated with nuclear reprogramming. Nat. Biotechnol. *27*, 353–360.

Do, J.T., Han, D.W., Gentile, L., Sobek-Klocke, I., Stehling, M., Lee, H.T., and Schöler, H.R. (2007). Erasure of cellular memory by fusion with pluripotent cells. Stem Cells *25*, 1013–1020.

Doege, C.A., Inoue, K., Yamashita, T., Rhee, D.B., Travis, S., Fujita, R., Guarnieri, P., Bhagat, G., Vanti, W.B., Shih, A., et al. (2012). Early-stage epigenetic modification during somatic cell reprogramming by Parp1 and Tet2. Nature *488*, 652–655.

Egli, D., Rosains, J., Birkhoff, G., and Eggan, K. (2007). Developmental reprogramming after chromosome transfer into mitotic mouse zygotes. Nature *447*, 679–685.

Feng, B., Ng, J.H., Heng, J.C., and Ng, H.H. (2009). Molecules that promote or enhance reprogramming of somatic cells to induced pluripotent stem cells. Cell Stem Cell *4*, 301–312.

Feng, S., Jacobsen, S.E., and Reik, W. (2010). Epigenetic reprogramming in plant and animal development. Science *330*, 622–627.

Ficz, G., Branco, M.R., Seisenberger, S., Santos, F., Krueger, F., Hore, T.A., Marques, C.J., Andrews, S., and Reik, W. (2011). Dynamic regulation of 5-hydroxymethylcytosine in mouse ES cells and during differentiation. Nature *473*, 398–402.

Fluckiger, A.C., Marcy, G., Marchand, M., Négre, D., Cosset, F.L., Mitalipov, S., Wolf, D., Savatier, P., and Dehay, C. (2006). Cell cycle features of primate embryonic stem cells. Stem Cells *24*, 547–556.

Foshay, K.M., Looney, T.J., Chari, S., Mao, F.F., Lee, J.H., Zhang, L., Fernandes, C.J., Baker, S.W., Clift, K.L., Gaetz, J., et al. (2012). Embryonic stem cells induce pluripotency in somatic cell fusion through biphasic reprogramming. Mol. Cell *46*, 159–170.

Fujii-Yamamoto, H., Kim, J.M., Arai, K., and Masai, H. (2005). Cell cycle and developmental regulations of replication factors in mouse embryonic stem cells. J. Biol. Chem. *280*, 12976–12987.

Ganier, O., Bocquet, S., Peiffer, I., Brochard, V., Arnaud, P., Puy, A., Jouneau, A., Feil, R., Renard, J.P., and Méchali, M. (2011). Synergic reprogramming of mammalian cells by combined exposure to mitotic Xenopus egg extracts and transcription factors. Proc. Natl. Acad. Sci. USA *108*, 17331–17336.

Ghule, P.N., Medina, R., Lengner, C.J., Mandeville, M., Qiao, M., Dominski, Z., Lian, J.B., Stein, J.L., van Wijnen, A.J., and Stein, G.S. (2011). Reprogramming the pluripotent cell cycle: restoration of an abbreviated G1 phase in human induced pluripotent stem (iPS) cells. J. Cell. Physiol. *226*, 1149–1156.

Gu, T.P., Guo, F., Yang, H., Wu, H.P., Xu, G.F., Liu, W., Xie, Z.G., Shi, L., He, X., Jin, S.G., et al. (2011). The role of Tet3 DNA dioxygenase in epigenetic reprogramming by oocytes. Nature *477*, 606–610.

Gurdon, J.B. (1960). The developmental capacity of nuclei taken from differentiating endoderm cells of Xenopus laevis. J. Embryol. Exp. Morphol. *8*, 505–526.

Gurdon, J.B., and Melton, D.A. (2008). Nuclear reprogramming in cells. Science *322*, 1811–1815.

Han, D.W., Do, J.T., Gentile, L., Stehling, M., Lee, H.T., and Schöler, H.R. (2008). Pluripotential reprogramming of the somatic genome in hybrid cells occurs with the first cell cycle. Stem Cells *26*, 445–454.

Hanna, J., Saha, K., Pando, B., van Zon, J., Lengner, C.J., Creyghton, M.P., van Oudenaarden, A., and Jaenisch, R. (2009). Direct cell reprogramming is a stochastic process amenable to acceleration. Nature *462*, 595–601.

Huangfu, D., Maehr, R., Guo, W., Eijkelenboom, A., Snitow, M., Chen, A.E., and Melton, D.A. (2008). Induction of pluripotent stem cells by defined factors is greatly improved by small-molecule compounds. Nat. Biotechnol. *26*, 795–797.

Inoue, A., and Zhang, Y. (2011). Replication-dependent loss of 5-hydroxymethylcytosine in mouse preimplantation embryos. Science *334*, 194.

Johnson, R.T., and Rao, P.N. (1970). Mammalian cell fusion: induction of premature chromosome condensation in interphase nuclei. Nature *226*, 717–722.

Koledova, Z., Kafkova, L.R., Calabkova, L., Krystof, V., Dolezel, P., and Divoky, V. (2010a). Cdk2 inhibition prolongs G1 phase progression in mouse embryonic stem cells. Stem Cells Dev. *19*, 181–194.

Koledova, Z., Krämer, A., Kafkova, L.R., and Divoky, V. (2010b). Cell-cycle regulation in embryonic stem cells: centrosomal decisions on self-renewal. Stem Cells Dev. *19*, 1663–1678.

Matveeva, N.M., Shilov, A.G., Kaftanovskaya, E.M., Maximovsky, L.P., Zhelezova, A.I., Golubitsa, A.N., Bayborodin, S.I., Fokina, M.M., and Serov, O.L. (1998). In vitro and in vivo study of pluripotency in intraspecific hybrid cells obtained by fusion of murine embryonic stem cells with splenocytes. Mol. Reprod. Dev. *50*, 128–138.

McNairn, A.J., and Gilbert, D.M. (2003). Epigenomic replication: linking epigenetics to DNA replication. Bioessays *25*, 647–656.

Mikkelsen, T.S., Hanna, J., Zhang, X., Ku, M., Wernig, M., Schorderet, P., Bernstein, B.E., Jaenisch, R., Lander, E.S., and Meissner, A. (2008). Dissecting direct reprogramming through integrative genomic analysis. Nature *454*, 49–55.

Miller, R.A., and Ruddle, F.H. (1976). Pluripotent teratocarcinoma-thymus somatic cell hybrids. Cell *9*, 45–55.

Neganova, I., and Lako, M. (2008). G1 to S phase cell cycle transition in somatic and embryonic stem cells. J. Anat. *213*, 30–44.

Niehrs, C., and Schäfer, A. (2012). Active DNA demethylation by Gadd45 and DNA repair. Trends Cell Biol. *22*, 220–227.

Orford, K.W., and Scadden, D.T. (2008). Deconstructing stem cell self-renewal: genetic insights into cell-cycle regulation. Nat. Rev. Genet. *9*, 115–128.

Papp, B., and Plath, K. (2011). Reprogramming to pluripotency: stepwise resetting of the epigenetic landscape. Cell Res. *21*, 486–501.

Pereira, C.F., and Fisher, A.G. (2009). Heterokaryon-based reprogramming for pluripotency. Curr. Protoc. Stem Cell Biol., *Chapter 4*, Unit 4B.1.

Pereira, C.F., Terranova, R., Ryan, N.K., Santos, J., Morris, K.J., Cui, W., Merkenschlager, M., and Fisher, A.G. (2008). Heterokaryon-based reprogramming of human B lymphocytes for pluripotency requires Oct4 but not Sox2. PLoS Genet. *4*, e1000170.

Pereira, C.F., Piccolo, F.M., Tsubouchi, T., Sauer, S., Ryan, N.K., Bruno, L., Landeira, D., Santos, J., Banito, A., Gil, J., et al. (2010). ESCs require PRC2 to direct the successful reprogramming of differentiated cells toward pluripotency. Cell Stem Cell *6*, 547–556.

Piccolo, F.M., Pereira, C.F., Cantone, I., Brown, K., Tsubouchi, T., Soza-Ried, J., Merkenschlager, M., and Fisher, A.G. (2011). Using heterokaryons to understand pluripotency and reprogramming. Philos. Trans. R. Soc. Lond. B Biol. Sci. *366*, 2260–2265.

Piccolo, F.M., Bagci, H., Brown, K., Landeira, D., Soza-Reid, J., Feytout, A., Mooijman, D., Hajkova, P., Leitch, H.G., and Tada,, T. (2013). Different roles for Tet1 and Tet2 proteins in reprogramming-mediated erasure of imprints induced by EG cell fusion. Mol. Cell X. Published online February 28, 2013. http://dx.doi.org/10.1016/j.molcel.2013.01.032.

Rao, P.N., and Johnson, R.T. (1970). Mammalian cell fusion: studies on the regulation of DNA synthesis and mitosis. Nature *225*, 159–164.

Ruiz, S., Panopoulos, A.D., Herrerías, A., Bissig, K.D., Lutz, M., Berggren, W.T., Verma, I.M., and Izpisua Belmonte, J.C. (2011). A high proliferation rate is required for cell reprogramming and maintenance of human embryonic stem cell identity. Curr. Biol. *21*, 45–52.

Savatier, P., and Afanassieff, M. (2002). [Cell cycle control and self-renewal of embryonic stem cells]. J. Soc. Biol. *196*, 117–123.

Selig, S., Okumura, K., Ward, D.C., and Cedar, H. (1992). Delineation of DNA replication time zones by fluorescence in situ hybridization. EMBO J. *11*, 1217–1225.

Serov, O.L., Matveeva, N.M., and Khabarova, A.A. (2011). Reprogramming mediated by cell fusion technology. Int. Rev. Cell. Mol. Biol. *291*, 155–190.

Silva, J., Chambers, I., Pollard, S., and Smith, A. (2006). Nanog promotes transfer of pluripotency after cell fusion. Nature *441*, 997–1001.

Simonsson, S., and Gurdon, J. (2004). DNA demethylation is necessary for the epigenetic reprogramming of somatic cell nuclei. Nat. Cell Biol. *6*, 984–990.

Singh, A.M., and Dalton, S. (2009). The cell cycle and Myc intersect with mechanisms that regulate pluripotency and reprogramming. Cell Stem Cell *5*, 141–149.

Soza-Ried, J., and Fisher, A.G. (2012). Reprogramming somatic cells towards pluripotency by cellular fusion. Curr. Opin. Genet. Dev. *22*, 459–465.

Stead, E., White, J., Faast, R., Conn, S., Goldstone, S., Rathjen, J., Dhingra, U., Rathjen, P., Walker, D., and Dalton, S. (2002). Pluripotent cell division cycles are driven by ectopic Cdk2, cyclin A/E and E2F activities. Oncogene *21*, 8320–8333.

Sullivan, S., Pells, S., Hooper, M., Gallagher, E., and McWhir, J. (2006). Nuclear reprogramming of somatic cells by embryonic stem cells is affected by cell cycle stage. Cloning Stem Cells *8*, 174–188.

Tada, M., Tada, T., Lefebvre, L., Barton, S.C., and Surani, M.A. (1997). Embryonic germ cells induce epigenetic reprogramming of somatic nucleus in hybrid cells. EMBO J. *16*, 6510–6520.

Tada, M., Takahama, Y., Abe, K., Nakatsuji, N., and Tada, T. (2001). Nuclear reprogramming of somatic cells by in vitro hybridization with ES cells. Curr. Biol. *11*, 1553–1558.

Takahashi, K., and Yamanaka, S. (2006). Induction of pluripotent stem cells from mouse embryonic and adult fibroblast cultures by defined factors. Cell *126*, 663–676.

Tat, P.A., Sumer, H., Pralong, D., and Verma, P.J. (2011). The efficiency of cell fusion-based reprogramming is affected by the somatic cell type and the in vitro age of somatic cells. Cell Reprogram. *13*, 331–344.

Terranova, R., Pereira, C.F., Du Roure, C., Merkenschlager, M., and Fisher, A.G. (2006). Acquisition and extinction of gene expression programs are separable events in heterokaryon reprogramming. J. Cell Sci. *119*, 2065–2072.

Theunissen, T.W., van Oosten, A.L., Castelo-Branco, G., Hall, J., Smith, A., and Silva, J.C. (2011). Nanog overcomes reprogramming barriers and induces pluripotency in minimal conditions. Curr. Biol. *21*, 65–71.

White, J., and Dalton, S. (2005). Cell cycle control of embryonic stem cells. Stem Cell Rev. *1*, 131–138.

Wu, H., and Zhang, Y. (2011). Mechanisms and functions of Tet protein-mediated 5-methylcytosine oxidation. Genes Dev. *25*, 2436–2452.

Xu, Y., Wu, F., Tan, L., Kong, L., Xiong, L., Deng, J., Barbera, A.J., Zheng, L., Zhang, H., Huang, S., et al. (2011). Genome-wide regulation of 5hmC, 5mC, and gene expression by Tet1 hydroxylase in mouse embryonic stem cells. Mol. Cell *42*, 451–464.

Yamanaka, S., and Blau, H.M. (2010). Nuclear reprogramming to a pluripotent state by three approaches. Nature *465*, 704–712.

Yang, V.S., Carter, S.A., Hyland, S.J., Tachibana-Konwalski, K., Laskey, R.A., and Gonzalez, M.A. (2011). Geminin escapes degradation in G1 of mouse pluripotent cells and mediates the expression of Oct4, Sox2, and Nanog. Curr. Biol. *21*, 692–699.

Yeom, Y.I., Fuhrmann, G., Ovitt, C.E., Brehm, A., Ohbo, K., Gross, M., Hübner, K., and Schöler, H.R. (1996). Germline regulatory element of Oct-4 specific for the totipotent cycle of embryonal cells. Development *122*, 881–894.

Supplemental Information

EXTENDED EXPERIMENTAL PROCEDURES

Centrifugal Elutriation of mESCs

Mouse ESCs (E14tg2A) were trypsinized and 3-5x10^8 resuspended in 10ml of elutriation buffer (1% FCS and 0.1% EDTA in PBS). Cells were gently passed through a syringe with an 18G needle and loaded into the chamber (Sanderson elutriation chamber; Beckman Coulter) at a flow rate of 6ml/min and a rotor speed of 1,800 r.p.m., 4°C. Subpopulations of cells were eluted by gradually increasing (1ml/min) the flow rate. For E14tg2A cells, enrichment of cells at G1, S or G2/M is achieved at 8-9ml/min, 12-14ml/min and 16-17ml/min, respectively. Slight variation in the cellular composition of fractions between experiments can occur as a consequence of the number of loaded cells and temperature of the elutriation media. When a standard chamber was used instead of a Sanderson chamber, centrifugation speed and flow rates were adjusted as suggested by the manufacturer (Beckman Coulter). At least 150ml was collected per fraction, and samples were collected into FCS, where appropriate, to enhance cell viability. To determine cell-cycle stages, cells were fixed in 70% Ethanol and their DNA content was assessed by propidium iodide (PI) staining, using FACScalibur (BD Biosciences) and CellQuest software.

Heterokaryons Immunofluorescence Assays

Heterokaryon immunofluorescence analysis used the following approach; elutriated and unsynchronized mouse ESCs were washed twice with PBS, resuspended in KO-DMEM medium supplemented with 10% FCS and plated in 0.1% gelatin coated coverslips. Once mouse ESCs attached, hB cells (previously washed twice with PBS) were gently resuspended in 1 ml of 37°C pre-warmed PEG and poured over the ESCs monolayer (mES:hB ratio 1:2). After 2 min incubation at 37°C, 2 ml of serum-free KO-DMEM was added and cells were centrifuged at 1,500 rpm for 5 min. Fused cells were cultured in KO-DMEM plus 2% horse serum and LIF for 3hr at 37°C. Finally, the medium was removed and cells were cultured under conditions promoting the maintenance of undifferentiated mouse ES cells.

Protein Extraction from Nuclear and Cytoplasmic Fractions

Cell pellets were resuspended in ice-cold lysis buffer (10mM HEPES, pH 7.9, 10mM KCl, 1.5mM $MgCl_2$, 0.34M Sucrose, 1mM DTT, 0.1% Triton X, 1.5mM PMSF and Protease inhibitors cocktail (Roche)) for 5 min on ice and centrifuged at 1,300 g for 5 min, 4°C. The cytoplasmic fraction (supernatant) was collected, washed once with lysis buffer without Triton-X and centrifuged for 10 min at 14,000 g, 4°C. The nuclear pellet was resuspended in nuclear lysis buffer (3mM EDTA, 0.2mM EGTA, 1mM DTT, 1.5 mM PMSF and Protease inhibitors cocktail (Roche)), incubated for 30 min on ice and centrifuged at 1,700 g for 5 min, 4°C. The pellet was washed once in nuclear lysis buffer and resuspended in 2x Laemmli buffer. The extracts were sonicated 3 x for 10 s each at 4°C, gently vortexed, incubated for 5 min at 95°C and chilled on ice. Protein extracts from 0.15×10^6 cells were loaded per lane.

Primers Used in Bisulfite Sequencing Analysis

Bisulfite-converted genomic DNA was used as template to amplify the endogenous (*Oct4*) and transgenic (GOF18ΔPE) (t*Oct4*) *Oct4* locus using the following primers: *Oct4* forward, 5′- TGGGTTGAAATATTGGGTTTATTT-3′ and reverse, 5′-TGGGTTGAAATATT GGGTTTATTT-3′; t*Oct4* forward, 5′-GGGGTTAGAGGTTAAGGTTAGAGG-3′ and reverse, 5′- ACCAAAATAAACACCACCCC-3′.

Primers Used in HpaII Resistance Assay

HpaII resistant fragments were quantified by qPCR using species-specific primers that flanked a HpaII/MspI sensitive site in the promoter region of *OCT4*. HpaII-sensitive site forward, 5′-GTGTCTGTGGAAGGGGAAAA-3′ and reverse, 5′-AGTTTCTGTGGGG GACCTG-3′; HpaII-insensitive site forward, 5′-CCACTAGCCTTGACCTCTGG-3′ and reverse, 5′- CCACCATTAGGCAAACATCC-3′.

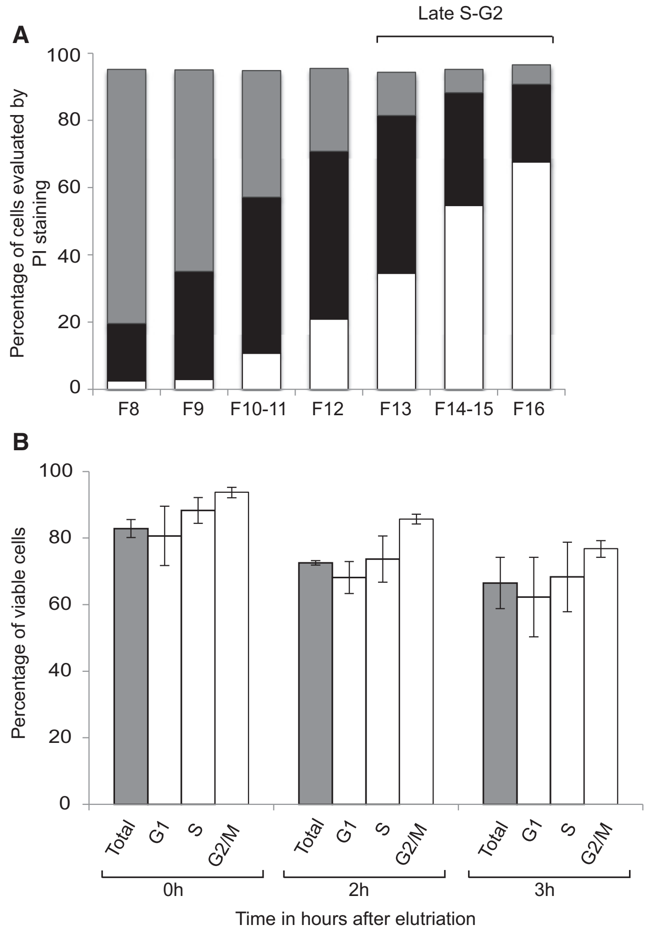

Figure S1. Enrichment and Viability of Mouse ESCs after Cell-Cycle Separation Using Counterflow Centrifugal Elutriation, Related to Figure 1
(A) Histograms show the proportion of ESCs corresponding to G1 (gray), S (black) and G2 (white), summarizing 5 independent elutriation experiments. The PI-staining profiles and gates used to define cells at different cell-cycle stages are indicated in Figure 1A.
(B) Unsynchronized (gray histograms) or ESCs enriched for G1, S or G2/M phases of the cell cycle (open histograms) were cultured for up to 3 hr in suspension in media containing LIF and viability was analyzed using trypan blue exclusion. Results show the mean and SD of four independent experiments.

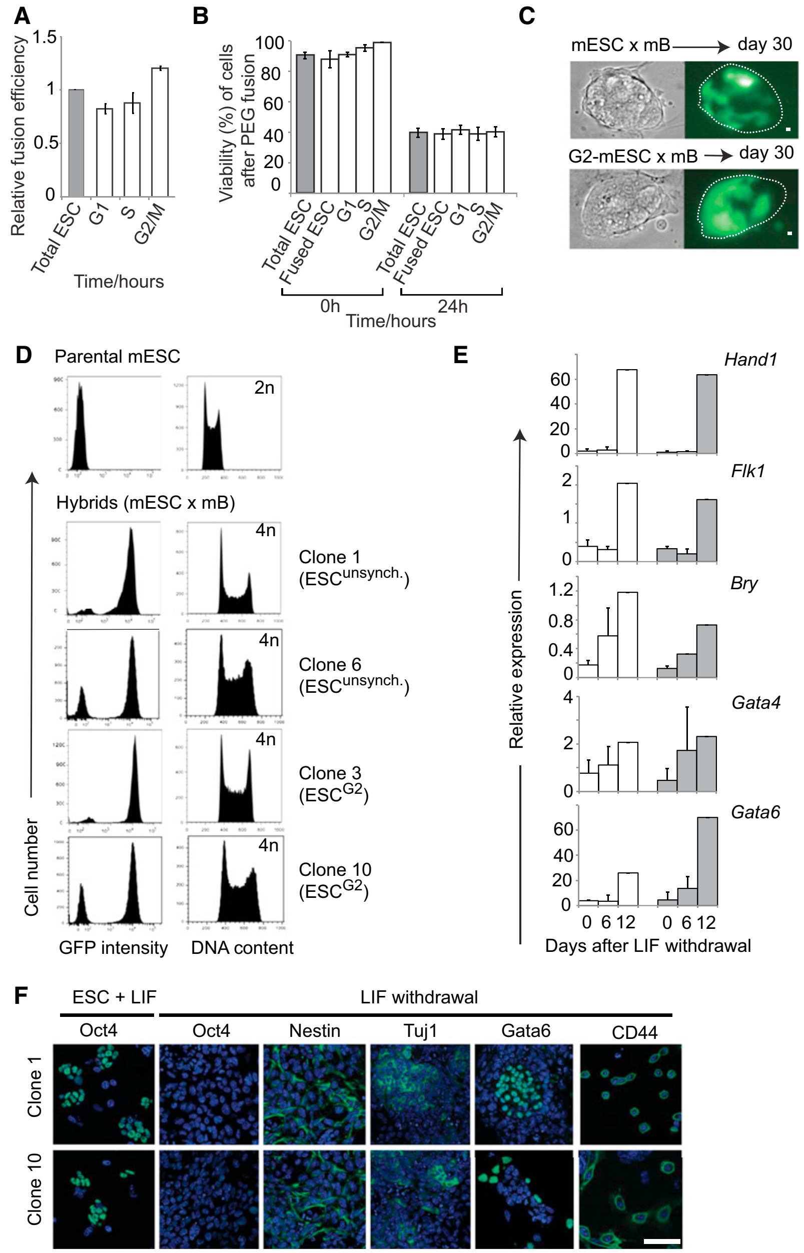

Figure S2. Characterization of Reprogrammed mB × mESC Hybrid Clones, Related to Figure 2

(A) Fusion efficiency was determined using H2B-mCherry mouse ESCs and human B cells labeled with carboxylfluorescein diacetate succinimidyl ester (CFSE). The frequency of double labeled cells was determined by FACS analysis and the fusion efficiency of different ESC cell-cycle fractions (open histograms) relative to unsynchronized samples (gray histogram) are indicated. Mean and SD of 3 independent experiments are shown.

(B) The viability of cells immediately after elutriation and 24 hr after PEG-mediated cell fusion was compared by trypan blue exclusion. Results show the mean and SD of 3 independent experiments.

(C) Hybrid reprogrammed clones were generated by fusing puromycin-resistant mB cells (derived from GOF18ΔPE) with G2-enriched or asynchronized ESCs. Colonies of puromycin resistant, GFP^{+} reprogrammed cells were isolated and expanded for 30 days in culture.

(D) DNA content and GFP expression was assessed by FACS. Metaphase spread analysis of these clones indicated similar average chromosome content (76 to 80), consistent with a broadly tetraploid status.

(E) Hybrid clones generated with unsynchronized (open histograms) or G2-enriched ESCs (gray histograms) were subjected to LIF withdrawal and the expression of several differentiation–associated genes was confirmed by qPCR. Error bars indicate the SD of two to three independent experiments.

(F) Confocal images showing the expression of pluripotent (Oct4), or of ectodermal (Nestin, Tuj1), endodermal (Gata6) and mesodermal (CD44) associated genes by hybrid clones (generated with unsynchronized or G2-enriched ESCs) before (ESC+LIF) or after LIF withdrawal for 10 days. Scale bar, 100 μm.

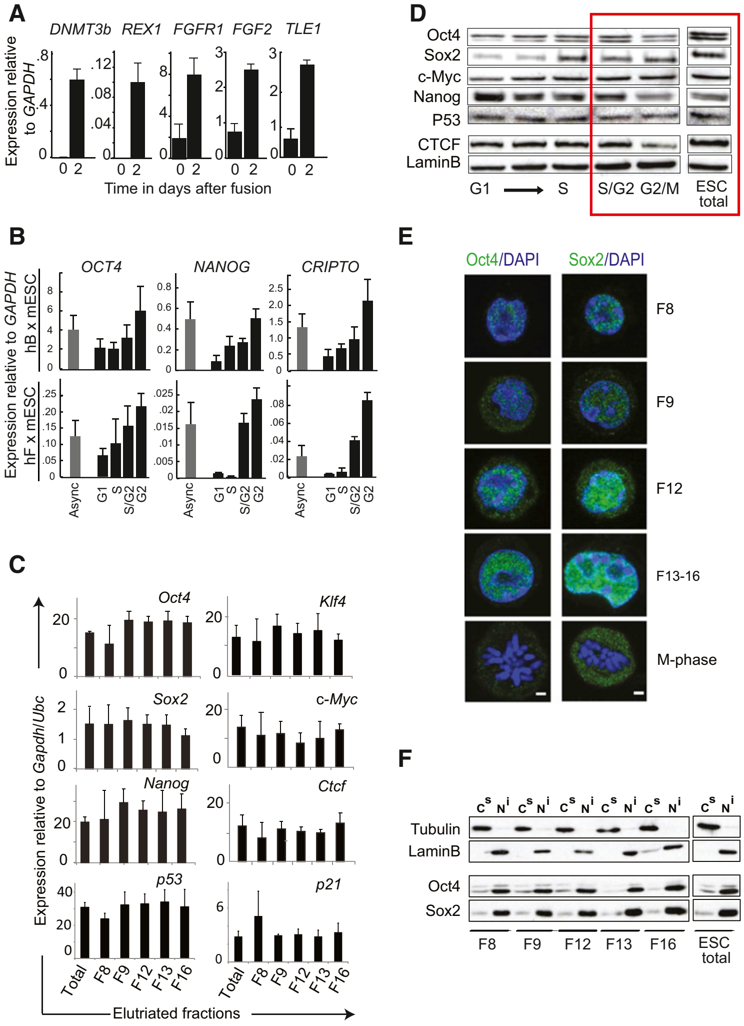

Figure S3. Characterization of Elutriated mESC Fractions, Related to Figure 3

(A) Induction of human pluripotency-associated genes was confirmed in heterokaryons formed between human B cells and G2-enriched mouse ESCs. This expression profile was similar to heterokaryons generated with ESCs (previously shown by Pereira et al. [2008]). Error bars indicate the SD of three independent experiments.

(B) Induction of human *OCT4*, *NANOG* and *CRIPTO* transcripts from human B (day 3) and human Fibroblasts (day 4) was improved by fusing with S/G2 and G2-enriched mouse ESCs, as compared with either asynchronous or G1-enriched fractions. Gene expression was calculated relative to *GAPDH* and values represent data from 2-4 independent experiments.

(C) Mouse pluripotency-associated gene transcript levels are similar throughout ESC cell-cycle progression. Quantitative RT-PCR analysis of the relative levels of gene expression in unsynchronized mouse ESCs and elutriated fractions enriched in G1 (F8-F9), S (F12), S/G2 (F13) or G2/M (F16). Gene expression data was normalized to *Gapdh* and *Ubc* and error bars indicate the SD of 4-5 independent experiments.

(D) Western blot analysis of mouse ESC extracts of unsynchronized (ESC, total) and populations enriched at different stages of the cell cycle (Fractions F8, F9, F12, F13, F16) in which antibodies to Oct4, Sox2 and c-Myc reveal the accumulation of Oct4 and Sox2 proteins at late stages of the cell cycle. CTCF and Lamin-B were used as loading controls. The red box enclose fractions with greater reprogramming potential to facilitate their comparison to extracts of unsynchronized ESCs.

(E) Confocal images of mouse ESCs showing the cellular distribution of Oct4 and Sox2 proteins (in green) during the cell cycle. Samples were separated by centrifugal elutriation (Fractions F8, F9, F12 and F13-16), subjected to IF and examined using identical laser settings for comparison. Among cells in fractions F13-16, 60% and 89% of cells showed moderate to intense labeling for Oct4 and Sox2, respectively. Background levels of Oct4 and Sox2 staining were seen in mitotic cells (15% and 12% of sample respectively). Nuclear DNA was stained with DAPI (blue) and scale bar, 5 μm.

(F) Western blot analysis of nuclear insoluble (N^i) and cytoplasmic soluble (C^s) extracts of unsynchronized ESCs and samples separated by counterflow centrifugal elutriation, using antibodies to Oct4 and Sox2. Oct4 and Sox2 proteins remain nuclear and chromatin-bound throughout interphase, and their abundance increases in samples enriched for late stages of the ESC cell cycle. Tubulin and Lamin-B were used as loading controls for proteins associated with cytosolic (C^s) and nuclear fractions (N^i), respectively.

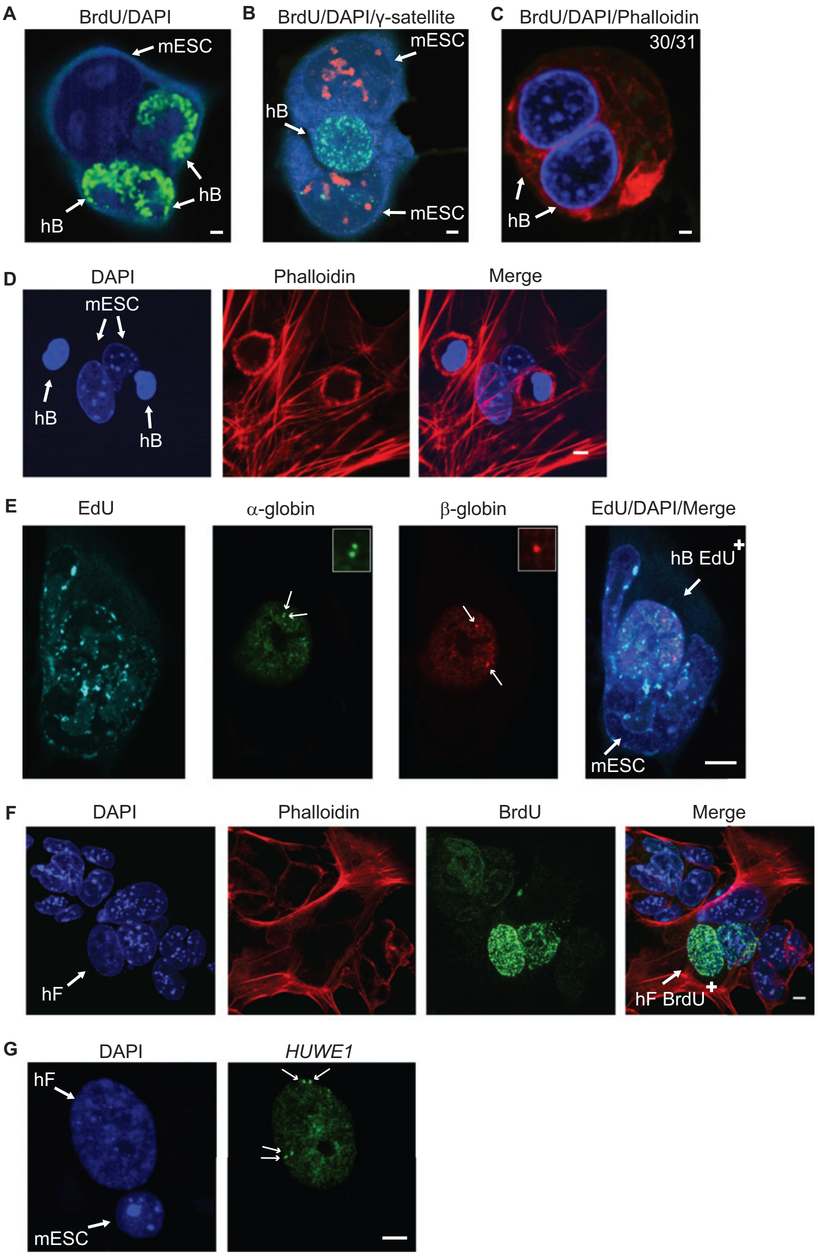

Figure S4. BrdU Labeling of Somatic Nuclei during Reprogramming, Related to Figure 4

(A) Confocal image of a mESC multikaryon (day 1) in which each of the 3 human nuclei showed similar BrdU-intense labeling pattern.

(B) Confocal Image of a representative heterokaryon pulse labeled with BrdU (green) 6 hr after PEG mediated cell fusion (hBxmESC) in which γ-satellite probe (red) was used to discriminate mouse ESCs. DAPI nuclear staining is shown in blue.

(C) Homokaryons formed between human B cells failed to show BrdU incorporation in most cells (30/31). Scale bars in A-C indicate 2μm.

(D) BrdU incorporation is not induced in hB cells that retain intact cell membranes after fusion with mESCs. A confocal image taken 24 hr after fusion of hB and mESCs (1:1), in which some human B cells (DAPI-diffuse, small nuclei) were engulfed within mouse ESCs (punctate DAPI labeling of larger nuclei) but retain their discrete cellular membranes, revealed by Phalloidin stain (red). BrdU incorporation in these 'encapsulated' human B cells was not detected (0/108). Scale bar, 5μm.

(E) A representative confocal image showing a single Z-section of a hBxmESC heterokaryon (24 hr after fusion), pulsed with EdU (100 μM, 45 min prior to fixation) (white), and processed for DNA FISH analysis using probes for human α-globin (GG1, green) and β-globin (ME2.5, red) and DAPI (blue, merged image) as described previously (Brown et al., 2001). In the human nucleus both α-globin alleles appeared as doublet signals consistent with replication having occurred (the second allele is not shown here). Both β-globin signals (a late replicating locus) appeared as singlet signals. Scale bar, 5 μm.

(F) Confocal images taken 24 hr after fusion of human fibroblasts (hF) and mESCs (in a 1:1 ratio) in which BrdU incorporation (green, a 45 min-pulse) by somatic hF nuclei (DAPI diffuse, arrow left) was detected (BrdU^{+}, arrow right). Scale bar, 5μm.

(G) Confocal images of a representative hFxmESC heterokaryon 72 hr after fusion, showing doublet signals for human *HUWE1* probe (green, arrowed) consistent with prior DNA replication (sample size > 100, scale bar, 5 μm).
